# The Effect of Digital Game-Based Learning on Learning Motivation and Performance Under Social Cognitive Theory and Entrepreneurial Thinking

**DOI:** 10.3389/fpsyg.2021.750711

**Published:** 2021-12-16

**Authors:** Chia-Chen Chen, Hsing-Ying Tu

**Affiliations:** Department of Management Information Systems, National Chung Hsing University, Taichung, Taiwan

**Keywords:** social cognitive theory, digital game-based learning, self-efficacy, competitive, entrepreneurship

## Abstract

This study aims to investigate the effects of students’ learning motivation and learning performance in a digital game-based learning setting and the structure of competition. This study uses Social Cognitive Theory, which emphasizes the bidirectional effects between personal factors, environmental factors, and behavior. We use the emotional state as the personal factor, social support as the environmental factor, learning performance as behavior. We also use self-efficacy and learning motivation as the mediating factors in the model. Data samples were collected from approximately 600 students in junior high schools in Taiwan. The students learned *via* either application or conventional lectures in three groups. The Control Group (CG) learned the course through a conventional learning approach. The Experimental group 1 (EG1) learned by a digital game, while Experimental Group 2 (EG2) learned through the digital game in combination with a structure that involved competing and entrepreneurship with classmates. The result of this research shows that the emotional state negatively affects learning motivation and self-efficacy, that self-efficacy will positively affect learning motivation, social support will positively affect self-efficacy, and self-efficacy and learning motivation will both positively affect learning performance. In addition, this research certifies previous works that entrepreneurs prefer to be more aggressive in competitions, have a high demand for accomplishment motivation, and are more likely to facilitate competitive over non-competitive environments.

## Introduction

Due to the rapid development of technology in the world, the Ministry of Education in Taiwan has developed new educational policies. In the “Curriculum Guidelines of 12-Year Basic Education” released by the Ministry of Education in 2014, the number of courses in the field of information technology was increased to cultivate students’ information literacy, information competitiveness, and their capability in terms of problem solving. However, in the past the main research subjects in Taiwan were examination subjects such as mathematics, English, and science. Few researchers have examined information technology courses. We wish to find the effect on students’ information literacy, competitiveness and provide educators in the field of computer science for reference through our research.

With the advance of science and technology, the teaching methods become diverse. Previous studies have researched the effect of students’ learning behavior in digital game-based learning environments worldwide (Lin et al., 2018; Chen, 2019; Lin et al., 2019; Wang et al., 2020). However, few studies in Taiwan have made the exploration on junior high school students’ learning environment and its relationship with learning motivation and performance in a digital game-based learning environment. To address this issue, this research develops a research model that explores personal, environmental, and behavior based on Social Cognition Theory (Bandura, 1989) with self-efficacy as the mediating factor.

To reduce the boredom of learning, teachers tend to integrate digital game-based learning into their curriculum. Over the past few years, digital games have been applied in a lot of subjects, such as mathematics (Hung et al., 2014), and science (Chen, 2019). Studies have shown that implementing digital game-based learning would increase students’ self-efficacy, learning motivation, and learning performance (Hung et al., 2014). Cagiltay et al. (2015) also showed that students’ learning performance would increase while integrating competition into digital game-based learning, yet studies in Taiwan rarely experiment with the conditions of competition in their research.

Many scholars outline that passion is “at the heart” of entrepreneurship (Baum, 2017; Obschonka et al., 2019). Entrepreneurial thinking needs a forceful passion that impacts personal agency, proactivity, creativity, risk-taking, aspiration, resilience, and persistence (Shen et al., 2021). In addition, entrepreneurial passion acts as an important factor in social interactions, especially for entrepreneurs who show passion and are aware of more success by investors, clients, and employees (Cardon and Kirk, 2015; Baum, 2017). Since entrepreneurial thinking is becoming more and more important for the success of organizations or enterprises, entrepreneurial passion is viewed as a critical factor for organizational behavior studies. To gain competitive advantages and ensure business survival in response to the external environment, innovation behaviors and their relation to entrepreneurship have received attention from scholars (Wu et al., 2019; Wei et al., 2020). In recent years, entrepreneurship education has become a major event in Higher Education Institutions around the world (Yueh et al., 2020). This education both increases students’ entrepreneurial skills and causes students to understand their personal characteristics and innovation behaviors as contributing to the efficacy of collaboration in an organization (Wu and Chen, 2019).

Based on the research motivation above, this study proposed two purposes below:

- To explore the effect of personal, environmental factors and learning motivations on students’ learning performance based on Social Cognitive Theory (SCT).
- To explore the effect of combining digital game-based learning and a competitive environment on students’ learning achievements and entrepreneurial thinking.

## Literature Review

### Social Cognitive Theory

**Figure 1:**
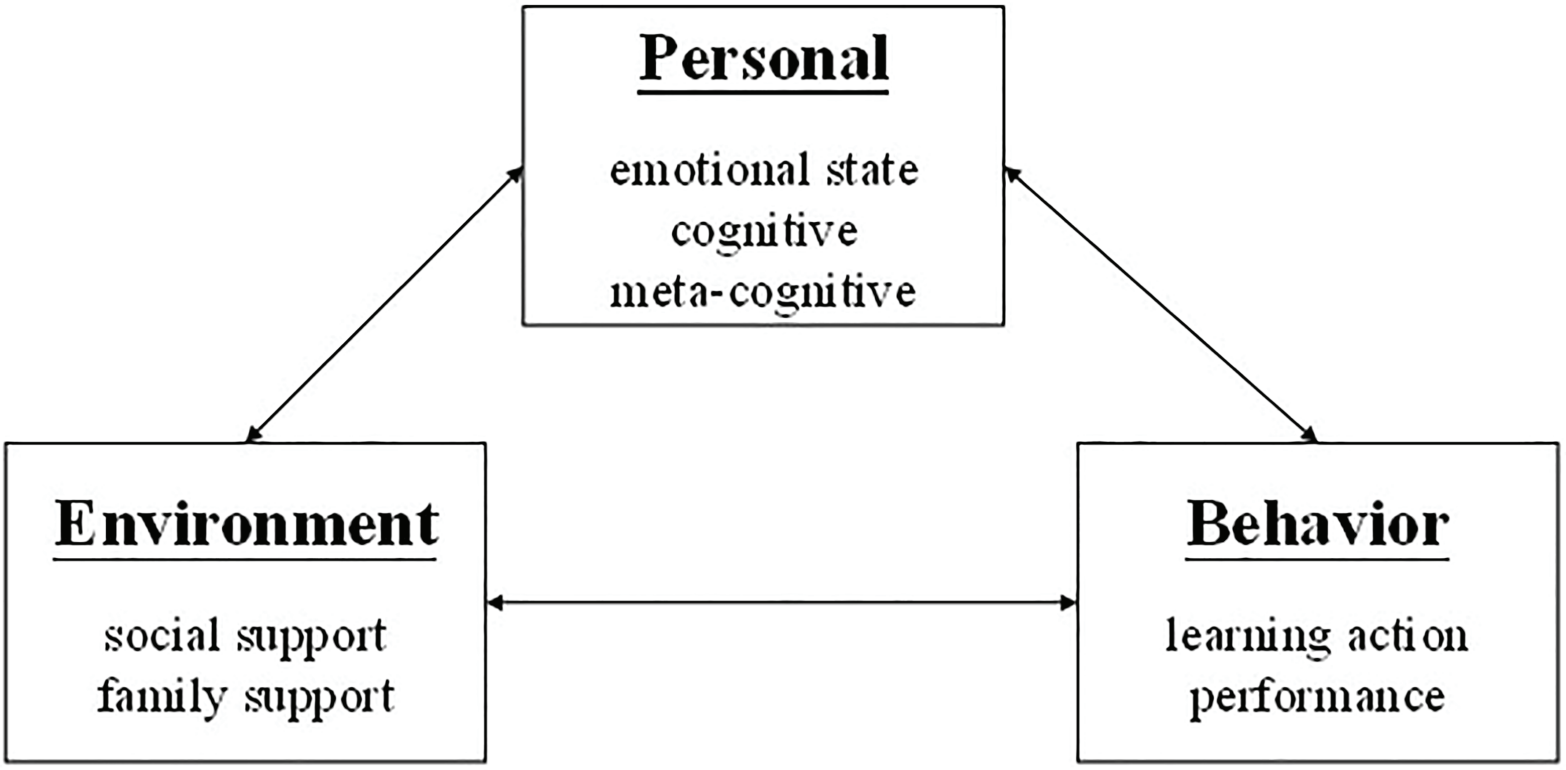
Social cognitive theory.

Before SCT, people considered that human behavior is affected by either personal or environmental factors, such as Behaviorism (Baum, 2017) or Dynamic Psychology (Woodworth, 1918). SCT was proposed by Bandura (1989). He believed a person’s abilities could be physically and psychologically different from each other due to their growth environment and social practice. SCT emphasized the bidirectional influence between personal, environmental factors, and behavior (Figure 1).

The relationship between personal factors and behavior means belief, thought, emotion, and action affect each other. According to this theory, the expectation of the future, a belief in one’s own abilities, goals, and intentions all lead to one’s behavior. What one thinks, feels, and believes will affect behavior. On the contrary, the perceptual system would be changed due to their behavioral experience. One’s expectation, belief, emotional orientation, and cognitive ability develop or adjust due to social influences. People also react differently to the social environment due to their physiological characteristics. For example, age, body shape, race, gender, and physical attractiveness could all cause great differences in people’s language and behavior. Likewise, a person’s different social reactions depend on their social role or status. In daily life, behavior will change environmental conditions, which in turn will change due to the conditions it creates. The environment is not necessarily a fixed entity that affects individuals. Most of the impacts in the environment must be triggered by appropriate behaviors to be effective. Unless the student participates in the course, the lecturer will not affect the student; parents usually do not praise their children unless they do something worthy of praise. Therefore, the potential environment’s impact on individuals must be transformed into actual impacts, which depend on their behavior (Bandura, 1989).

Self-efficacy is another important aspect of SCT. It was proposed by Albert Bandura in 1977. It refers to the degree of belief that people can successfully perform the behaviors required to produce results based on past experience or self-assessment. Their strength of beliefs will affect their reactions to problems or the situations they encounter. Therefore, self-efficacy will change people’s choices of behavior (Dissanayake et al., 2019).

Bandura (1977) indicated that there were four sources of self-efficacy, performance accomplishments, vicarious experience, verbal persuasion, and physiological/emotional state. Performance accomplishments are based on one’s past experience. Successful experience will increase one’s self-efficacy, while the failed experience will reduce their self-efficacy. Vicarious experience is self-inference after comparing with others. Verbal persuasion is used when we trying to change one’s behavior. People rely partly on their emotional or physical state to determine the degree of their anxiety and stress. Compared with nervous and anxious people, people who are not affected by their emotional or physiological state are more likely to be successful (Lin and Tsai, 2018).

Social Cognitive Theory has been applied in different subjects, such as health (Lin and Chang, 2018), jobs (Sheu et al., 2017), and education (Lin et al., 2018). Burnett et al. (2016) investigated students’ perceptions of cheating by applying SCT. They found that students chose to cheat because of the enrollment of graduate school or peer pressure. Wang et al. (2017) investigated the relationship between self-efficacy, social support, self-regulated learning, and learning motivation in a competitive environment. They discovered that all the factors would positively affect each other, except social support.

### Learning Motivation

Learning motivation refers to the motivation that triggers or maintains the learning behavior of students. It is the direct cause of learning. Through learning motivation, you can understand whether students want to learn, what they like to learn, and how hard they are willing to work for learning. Learning motivation is usually defined as intrinsic motivation and extrinsic motivation (Ryan and Deci, 2000).

Intrinsic motivation is inner satisfaction after completing a learning activity. When a person is intrinsically motivated, they join the learning activity because it is fun or a challenge, not because of pressure or the award. On the contrary, extrinsic motivation means students are motivated to learn because of external factors, such as students working hard to avoid punishment from their parents. Studies have shown the importance of learning motivation due to its effect on students’ learning performance (Liu et al., 2018).

### Digital Game-Based Learning

Digital game-based learning refers to learning by solving problems or accomplishing tasks through computers, mobile phones, or tablets. There are several elements in digital game-based learning, such as fun, play, goals, competition, and problem-solving (Sandberg et al., 2014). Studies have investigated digital game-based learning in different subjects, such as English (Yang and Chen, 2020), Maths (Hung et al., 2014; Deng et al., 2020), Sciences (Chen, 2019), and STEAM (Chen and Huang, 2020). The results indicate that digital game-based learning has gradually become a trend and that digital game-based learning could improve students’ learning motivation (Su, 2016; Lin et al., 2018), learning performance (Lin et al., 2018), reduce their cognitive load (Su, 2016; Chen and Huang, 2020), and anxiety (Su, 2016).

### Entrepreneurship and Competition

When two or more individuals struggle for an unachievable goal (status, recognition, prizes, etc.), there will be competition. Competition is the main factor in education. To promote the achievements of the next generation, a national education system encourages competitiveness among students through scholarships. In the past, scholars believed that competition would only have a negative impact on students. However, in recent research, it has been found that the impact of competition depends on students’ ability to act. Compared to students with low ability to act, students with high ability will grow up in competition and willing to take risks. These students are more likely to have flexibility, adaptability, and creativity in the future (Eisenberg and Thompson, 2011). Johnson and Johnson (2009) suggest that competition with clear, fair rules and procedures will help students improve their learning achievements. Studies have incorporated competition into research as a factor of discussion to stimulate students’ potential (Lim and Oliver, 2015) or maintain their confidence (Vogl and Preckel, 2014).

Studies have also integrated competition into game-based learning. There are several ways of implementing competition, for example, learners compete with themselves, the system, or other learners (Chen et al., 2019). Learners might improve their learning motivation, attention, effort, and excitement, etc. (Cagiltay et al., 2015). Some researchers believe that when learners see the scores of other competitors, they lose their learning motivation, or they only focus on winning against the others without actually learning (ter Vrugte et al., 2015).

According to research by Schumpeter (1934) and Kirzner (1973), entrepreneurship highly impacts competition and human behavior. They realized that there was little existing research about entrepreneurs and the psychological situations involved in a competitive environment. After that, Rauch and Frese (2007) investigated the personality characteristics of entrepreneurs and matched their specific issues through expert interviews. Niederle (2017) pointed out that behavioral economists face career choices and individual competitiveness. It means an individuals’ general tendency turns towards competitive over non-competitive situations. Meanwhile, some scholars also extended the investigation to more competitive educational and occupational environments (e.g., Almås et al., 2016; Buser et al., 2017; Reuben et al., 2017). Therefore, this research integrated perspectives of entrepreneurship and SCT to propose the research model and hypotheses development in the following section.

## Materials and Methods

### Hypotheses Development

Based on the literature review, this study selected three factors under SCT. We take social support as the factor of environment because junior high students were more likely influenced by their peers than their teachers or their parents, emotional state as the personal factor, and learning performance as the behavior factor. We even wanted to verify the effect between self-efficacy, learning motivation, and learning performance. The hypothesized relationships between SCT, self-efficacy, learning motivation, and learning performance are described and explained below.

#### Emotional State and Learning Motivation

Emotional state means the state of arousal characterized by alteration of feeling tone and by physiologic behavioral changes. Students tend to have higher learning motivation when they enjoy learning. On the other hand, when students feel nervous, depressed, or under pressure, they may have lower learning motivation. Arguedas et al. (2016) found that if students understand their emotions in learning, students’ learning motivation can be enhanced through emotions with the guidance of a specific strategy. Studies have shown that stress is negatively correlated with learning motivation, which means the greater the pressure, the lower their learning motivation (Johnsen et al., 2017). Some studies have also shown that anxiety and depression negatively affect students’ learning motivation (Su, 2016; Kunanitthaworn et al., 2018). Özhan and Kocadere (2020) discovered that if students enjoyed the class and paid attention, they would have higher learning motivation. Therefore, a hypothesis was developed:

*H1:* Emotional state is negatively related to learning motivation.

#### Self-Efficacy and the Factors Under SCT

Self-efficacy was proposed by Bandura (1977). It refers to students’ belief in their capacity to execute the behaviors necessary to produce specific performance attainments. An emotional state is one of the sources of self-efficacy. People with different emotional states have different self-efficacy. Students with negative emotions tend to have lower self-efficacy (García-Pérez et al., 2020). Hong et al. (2019a) discovered that if cognitive anxiety negatively affects self-efficacy, students’ belief in their ability would decrease when they felt anxious during their learning process. Pumptow and Brahm (2021) found that enjoyment positively affects self-efficacy, while anxiety negatively affects self-efficacy.

Liu and Hung (2016) found that students in the Republic of Columbia with higher social support have more self-efficacy. When people receive support from their peers, family, or teacher, they have higher self-efficacy and more confidence in their ability (Lent et al., 2016). The same results were found in Lent et al. (2017) and Sheu et al. (2017).

Some studies have shown that self-efficacy is positively related to learning performance. Joo et al. (2015) found that self-efficacy is positively associated with learning performance in research on online learning in Korea. In the research of GPS sensor-based mobile learning for English, Sun et al. (2015) found that students who have better English learning performance have higher self-efficacy. The results were also found in the research with different subjects, such as Mathematics (Tian et al., 2018) and Science (Uçar and Sungur, 2017). Therefore, three more hypotheses were developed:

*H2:* Emotional state is negatively related to self-efficacy.

*H5:* Social support is positively related to self-efficacy.

*H7:* Self-efficacy is positively related to learning performance.

#### Social Support and the Factors Under SCT

Emotional state plays an important role in learning and this study focuses on students’ stress, anxiety, and nervousness when in class. Studies have shown that positive factors were related to environmental support and that students that experience positive effects were more likely to gain support from their peers, family, or teachers (Liu and Hung, 2016; Lent et al., 2017). On the other hand, students who experience negative effects tend to have less support from the people around them (Franco et al., 2019).

Environmental support is defined as the support students receive from their peers, family, and teachers. This study focuses mostly on social support, which is the support they received from their peers. Students with more support were more likely to have better learning performance. King and Ganotice (2014) suggest that we could improve students’ learning performance by improving their social support. Oranye et al. (2017) both found that students with higher social support would have better learning performance. Therefore, to examine the effects between emotional state, social support, and learning performance, two hypotheses are developed:

*H3:* Emotional state is negatively related to social support.

*H8:* Social support is positively related to learning performance.

#### Self-Efficacy and Learning Motivation

Learning motivation was defined as an established pattern of pursuing goals, beliefs, and emotions (Ford, 1992). Previous research has indicated that self-efficacy has a significant positive effect on learning motivation (Tian et al., 2018; Pumptow and Brahm, 2021). Phan (2016) divided the learning process into six time periods, and it found that self-efficacy in the middle stage affects learning motivation in the later stage. Wang et al. (2017) found that self-efficacy significantly affects learning motivation in a CSCL environment with coopetition design. Dissanayake et al. (2019) found that self-efficacy would positively affect learning motivation in both competitive and non-competitive environments. Zhao and Huang (2020) found that students with high self-efficacy have high learning motivations. Zhan et al. (2021) found that self-efficacy and intrinsic motivation had a highly positive relationship, and discovered that self-efficacy and learning motivation could positively affect learning strategy. Therefore, another hypothesis was developed:

*H4:* Self-efficacy is positively related to learning motivation.

#### Learning Motivation and Learning Performance

Motivation is important in students’ learning. Studies have shown that learning motivation is positively related to learning performance. Students with higher learning motivation have higher learning performance (Su, 2016; Tian et al., 2018; Zhao and Huang, 2020). Tokan and Imakulata (2019) examined the influence of intrinsic motivation and extrinsic motivation on students’ learning behavior and learning performance in learning biology. They found that intrinsic motivation directly affected students’ learning behavior and learning performance. Zhao and Huang (2020) found that learning motivation and learning performance had a highly positive relationship. Therefore, another hypothesis is proposed:

*H6:* Learning motivation is positively related to learning performance.

**Figure 2:**
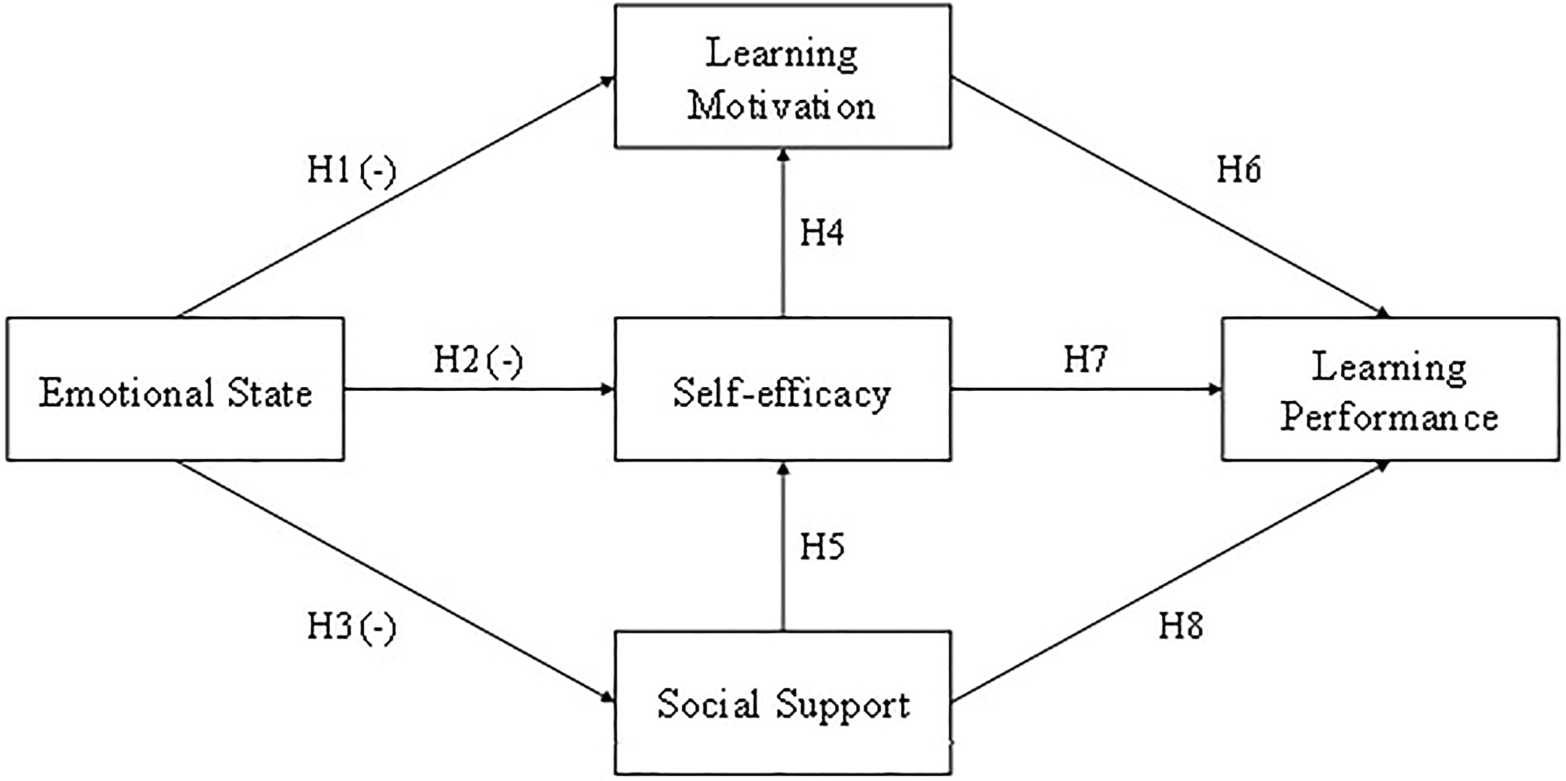
Research model.

A research model composed of all the hypotheses is shown in Figure 2.

### Hypothesis Testing Method

We used structural equation modeling (SEM) to test our research model (Figure 2). To ensure the study had good reliability and validity, preliminary tests, which involved checking the unidimensionality of the five parts of the construct (Emotional state, Social support, Self-efficacy, Learning motivation, Learning performance), were taken.

### Settings and Participants

**Figure 3:**
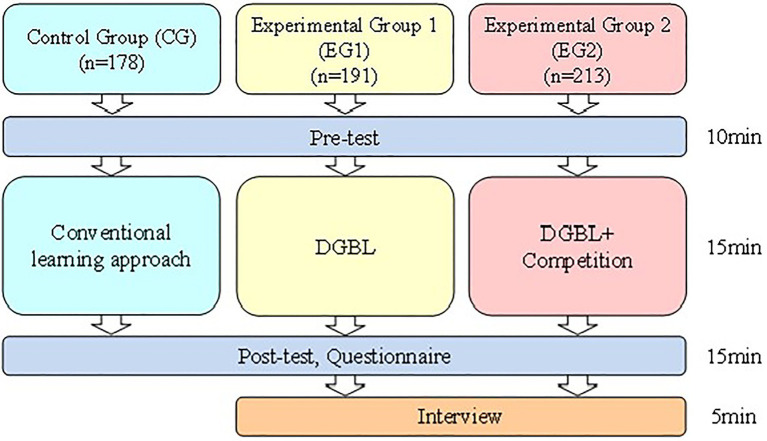
Experiment procedure.

Data were collected among junior high school students in Taichung, Taiwan. There were approximately 600 students. All the participants learned the basic concept of binary conversion. The experimental procedure was conducted for 50 min (Figure 3). External validity refers to the appropriateness of the dynamic relationship between the various variables in the research if it is taken by different populations, experimental conditions, or specific experimental period. To improve the external validity of the research, we took larger populations and random sampling. Therefore, this research collected approximately 600 students and also adopted random sampling for three different groups. The students learned *via* either application or conventional lectures in three groups. The control group (CG) learned the course through a conventional learning approach, which means the instructor would only use a textbook or PowerPoint to teach the course. Experimental Group 1 (EG1) could only learn by a digital game, while Experimental Group 2 (EG2) learned through a digital game, with the added structure of competing with classmates.

**Figure 4:**
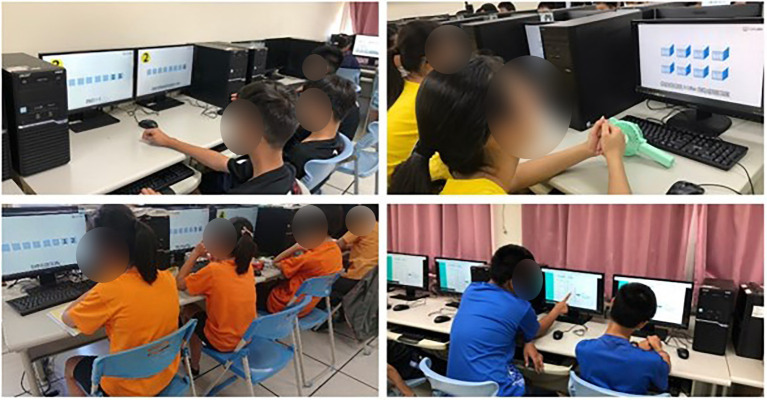
Learning activities for CG.

**Figure 5:**
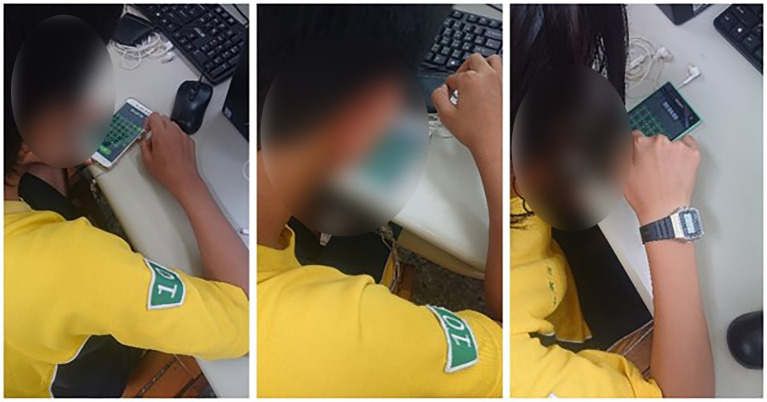
Learning activities for EG1.

**Figure 6:**
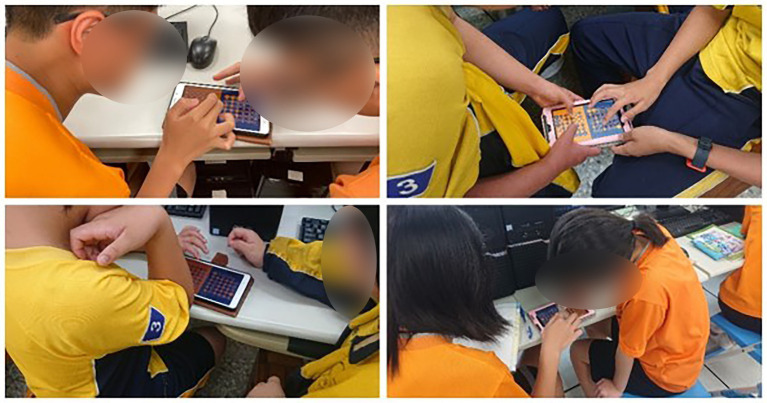
Learning activities for EG2.

All of the students took a knowledge exam before and after the experiment and the questionnaire was conducted post-test. The interview was conducted after the experiment with students in EG1 and EG2. The learning activities for the experimental procedure are shown in Figures 4–6.

### Measurement Instrument

The hypotheses were tested using quantitative research. We designed a list of questions correlated with the factors in our model (see Appendix 1). The first part consists of emotional state (ES) adapted from Usher and Pajares (2009), social support (SS) adapted from Cohen et al. (1985), self-efficacy (SE) adapted from the Motivated Strategies for Learning Questionnaire (MSLQ; Pintrich et al., 1991), learning motivation (LM) adapted from Glynn et al. (2011), and learning performance (LP) adapted from Li (2012). All of the items in the questionnaire were on a 5-point Likert scale. The second part consists of the personal particulars of respondents, such as gender, and their class.

A learning performance test, which examined the students’ binary conversion knowledge, was used for both the pre-test and the post-test. The test consisted of 10 questions, each worth 10 points. Two teachers developed the test for this study, and each teacher taught computer science in junior high school for 2 years. The questions of pre-test and post-test are different, but the difficulty was equivalent.

The interview was conducted by adopting Fidan and Tuncel (2019) to collect experimental group students’ perceptions and opinions of using the application to learn based on the following questions:

1. Do you think this method of using an application to learn binary conversion is fun? Why?
2. Do you think this method of using an application to learn binary conversion is more effective? Why?
3. Do you think this method of using an application to learn binary conversion could make learning computer science more interesting? Why?
4. Do you think this method of using an application to learn binary conversion could make you more confident in learning computer science? Why?

## Results

### Student Demographics

An overview of respondents is shown below in Supplementary Table 1. There were a total of 582 students and 510 valid samples. There were 48 invalid samples in EG1. The reason for this could be because most participants were in the ninth grade and had just finished their big exam for university, meaning they did not have the patience to finish the questionnaire. According to Hair et al. (1998), the respondent of the questionnaire should exceed five times the questions, and the number of valid samples should exceed 100. The number of questionnaires in this study was 22, meaning the minimum required samples was 110. The valid samples achieved the recommendation level outlined by Hair et al. (1998).

### The Measurement Model

The measurement model in SEM was evaluated in terms of reliability, convergence validity, discriminative validity, and model fit.

#### Reliability

This study used Cronbach’s *α* to determine the reliability of the construct and the value is shown below (Supplementary Table 2). The value of Cronbach’s *α* should be above 0.7 to show the reliability of the construct. Results showed that the Cronbach’s *α* of all constructs exceeded the criterion of recommended reliability suggested by Fornell and Larcker (1981), which means all the constructs have good reliability.

#### Convergence Validity

Convergence validity is used to test whether items in the same dimension can be constructed to represent most of their constructs, and eventually converge in the same dimension. We used the most commonly used factor loadings, composite reliability (CR), and average variance extracted (AVE) to test convergence validity. The value of all the items is shown below in Supplementary Table 3.

According to the suggestion of Fornell and Larcker (1981), the value of factor loadings should exceed 0.5 to show the sufficiency of representing the construct. The results showed that SS2 for social support, SE4 for self-efficacy received lower factor loadings than the suggested factor loadings value of 0.5 and were therefore eliminated. Based on the suggestion of Fornell and Larcker (1981), the value of CR should be above 0.6 to show great internal consistency in the construct. As shown in Supplementary Table 3, the CR of all the constructs was above 0.8, indicating all constructs had good internal consistency. The AVE should be above 0.5 based on the recommendation of Fornell and Larcker (1981). The AVE of all the constructs was above 0.5 in this study, as shown in Supplementary Table 3, which showed great convergence validity of the constructs.

#### Discriminative Validity

Discriminative validity is used to test the correlation between constructs to discriminate measures in dissimilar constructs. The value of the square roots of AVE and the inter-correlation are shown below (Supplementary Table 4). The inter-correlation value should be below the square roots of AVE and should not be above 0.85 based on the suggestion of Hair et al. (1998). Results showed that the inter-correlation of every construct met the suggestion, which indicates the good discriminative validity of the constructs.

#### Model Fit

Model fit is assessed using absolute fit indices, incremental fit indices, and parsimonious fit indices. There were a total of 12 index for examining the model. The value of all the index are shown below in Supplementary Table 5. Results showed that all the values of every index met the recommendation.

### The Structural Model

**Figure 7:**
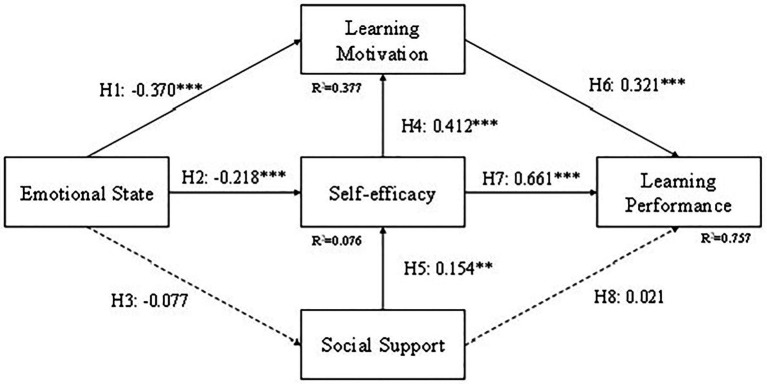
Model results. ^**^p < 0.01; ^***^p < 0.001.

We used structural model assessment to test the theoretical hypotheses we proposed, which included the relationship between emotional state, social support, self-efficacy, learning motivation, and learning performance (Figures 2, 7). We used the coefficient of determination (*R*^2^), path coefficients (*β*), and critical ratio (C.R.) to determine how well the data supported the relationship of the hypotheses. The results are shown in Figure 4, the structural path coefficients are shown in Supplementary Table 6, and the hypotheses justification is shown in Supplementary Table 7. The results in Supplementary Table 7 indicate that the pathways have significant causalities from Emotional state to Learning motivation (*β* = −0.370, C.R. = −8.692, *p* < 0.001; H1), Emotional state to Self-efficacy (*β* = −0.218, C.R. = −4.455, *p* < 0.001; H2), Self-efficacy to Learning motivation (*β* = 0.412, C.R. = 9.088, *p* < 0.001; H7), Social support to Self-efficacy (*β* = 0.154, C.R. = 3.009, *p* = 0.003 < 0.01; H3), Learning motivation to Learning performance (*β* = 0.321, C.R. = 8.662, *p* < 0.001; H8), Self-efficacy to Learning performance (*β* = 0.661, C.R. = 13.347, *p* < 0.001; H4). However, Emotional state to Social support (*β* = −0.077, C.R. = −1.501, *p* = 0.133; H5), Social support to Learning performance (*β* = 0.021, C.R. = 0.666, *p* = 0.506; H6) are not supported.

### Group Comparison

**Figure 8:**
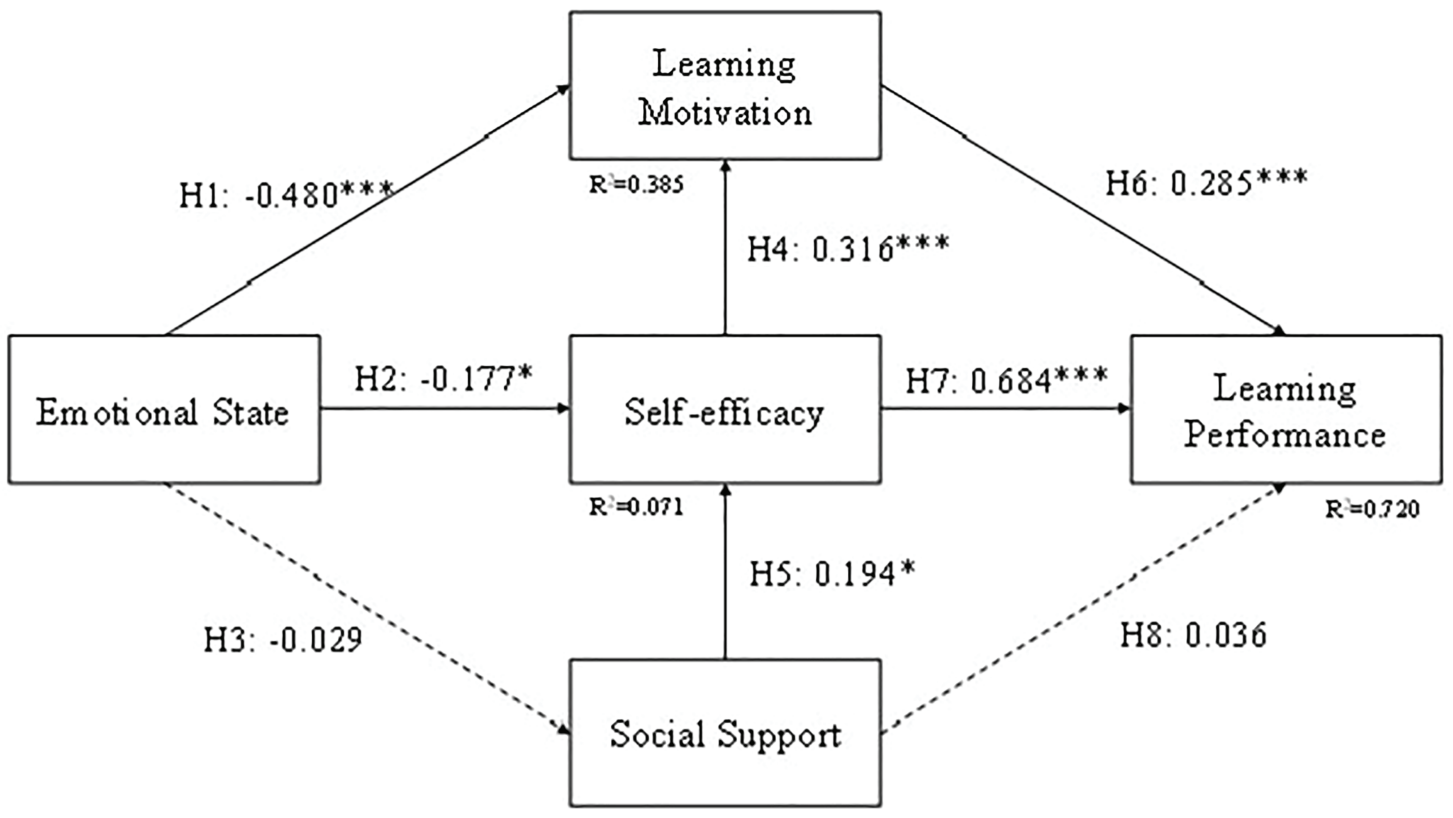
Model result for CG. ^*^p < 0.05; ^***^p < 0.001.

**Figure 9:**
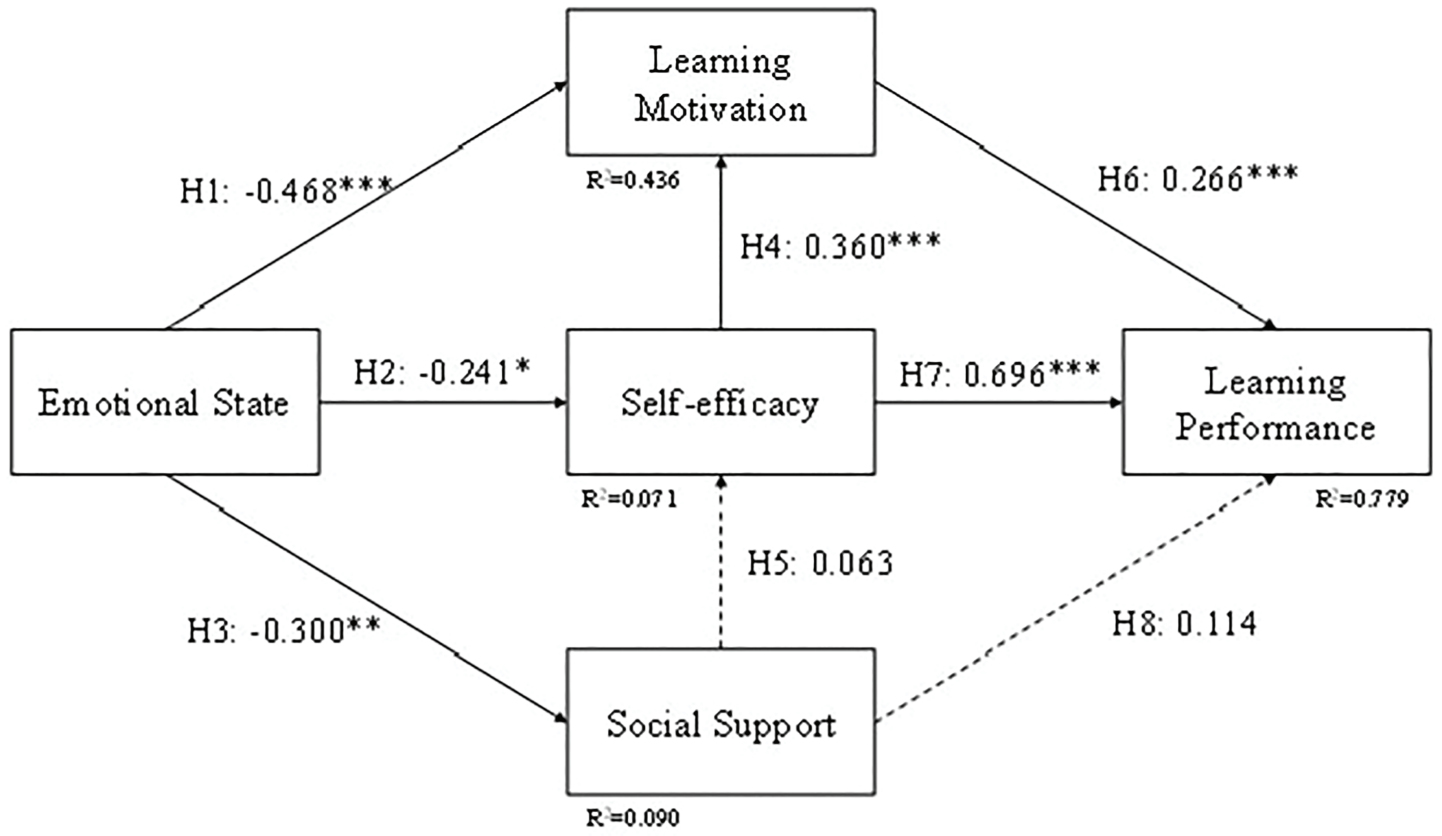
Model result for EG1. ^*^p < 0.05; ^**^p < 0.01; ^***^p < 0.001.

**Figure 10:**
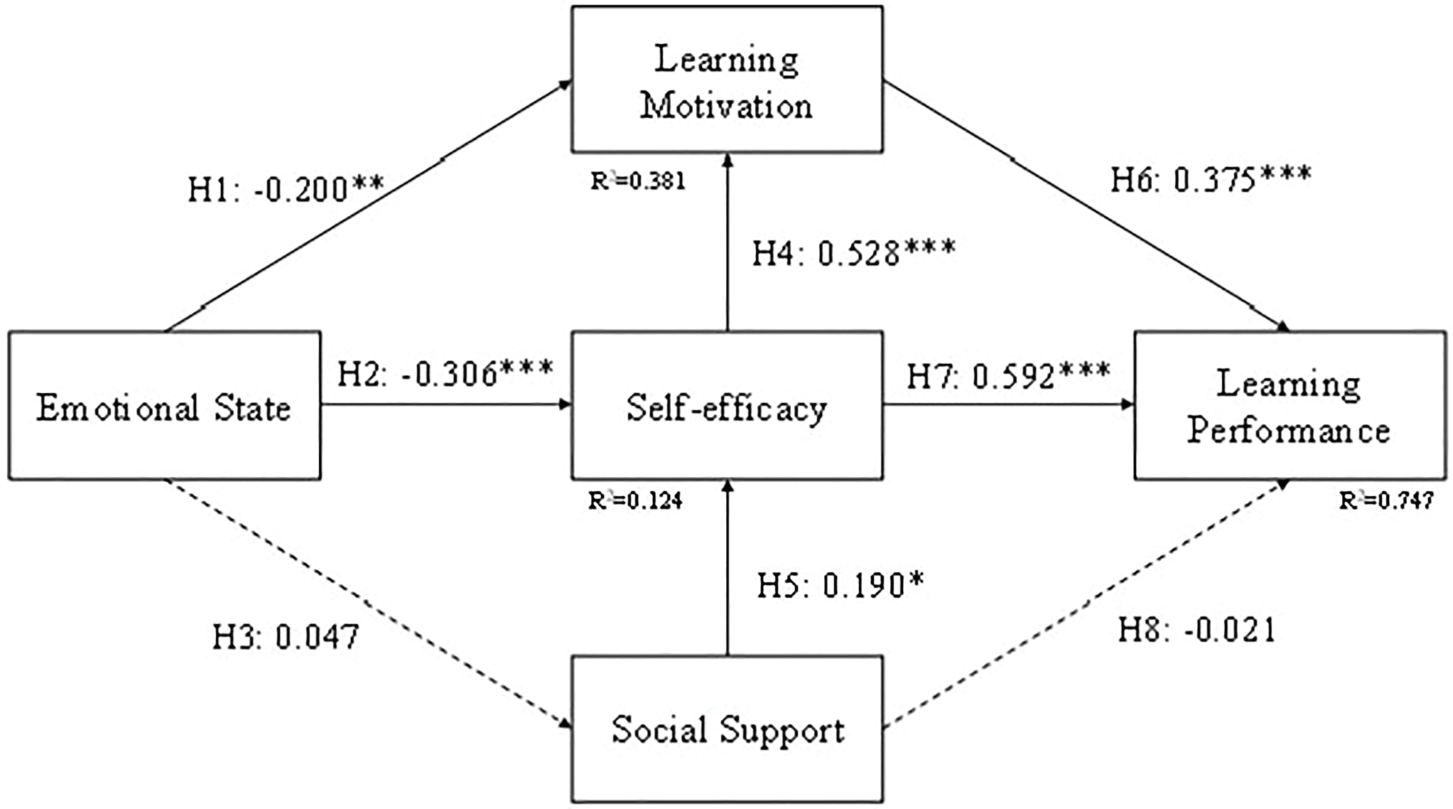
Model result for EG2. ^*^p < 0.05; ^**^p < 0.01; ^***^p < 0.001.

Students were divided into three groups, CG, EG1, and EG2. In order to compare the results between different teaching methods, we tested the structural model for these three groups and the results are shown in Figures 8–10. The difference in EG1 is that H5 (Emotional state to Social support) is supported and H3 (Social support to Self-efficacy) is not supported. The reason for H5 might be because students in EG1 were mostly in ninth grade and have a better relationship with their peers. They tend to share the excitement they acquired during learning with their friends compared to the other two groups. The reason for H3 might be that self-efficacy for students in EG1 might come from learning through a tablet or a cellphone but not their friends. Or it might be the emotional state caused by the curiosity of using a tablet or a cellphone, not by the assistance they received from their friends.

The results of CG and EG2 are the same as the structural model of all the students. The H5 (Emotional state to Social support) and H6 (Social support to Learning Performance) are not supported in CG and EG2. In the EG1 result, if the H5 (Emotional state to Social support) is supported then we can assume that game-based learning may have been a useful learning style for learners. However, as this research was designed with entrepreneurship and competition in mind, as indicated by EG2, then we can observe that entrepreneurship and individual competitiveness will have a negative impact on personal emotional state with social support.

### Learning Achievement

Students were divided into three groups to investigate the effect of the teaching methods in this study. We used ANOVA to investigate the effectiveness of the teaching method. The results of the pretest are shown in Supplementary Tables 8 and 9, and the results of the post-test are shown in Supplementary Tables 10 and 11.

According to the result in Supplementary Tables 8 and 9, the scores of learning achievement between CG, EG1, and EG2 are not significantly different (*p* > 0.05). This means that students’ prior knowledge of binary conversion in all three groups is at the same level. The result in Supplementary Table 10 showed that CG, EG1, and EG2 are significantly different (*p* < 0.001). Based on the result in Supplementary Table 11, CG and EG1 have a highly significant difference, CG and EG2 have an extremely significant difference, and EG1 and EG2 have significant differences. This indicates that, based on the same level of prior knowledge, students who studied digital game-based learning and in the environment of competition (EG2) have higher learning achievements. Students who learned with digital game-based learning (EG1, EG2) had higher learning achievements compared to students who learned with a conventional learning approach (CG).

### Interview

1. Do you think this method of using an application to learn binary conversion is fun? Why?

About 85% of students in EG1 expressed that digital game-based learning was interesting. Most of the students indicated that it was fun to learn new knowledge using this kind of teaching method. Some students thought the lessons become interesting because they were able to use cellphones and tablets to play games. Some students thought that it was not fun to calculate numbers, even if using digital game-based learning to learn binary conversion.

94% of students in EG2 thought that interacting with peers through the digital game is fun and novel and made learning much more interesting. Some students indicated that it was easier and enjoyable when they learned through the game. Few students found that they were burdened by the experience of competing with others.

2. Do you think this method of using an application to learn binary conversion is more effective? Why?

86% of students in EG1 thought that integrating digital games into the course enabled them to know binary conversion better, and that it was easier to learn. Some students replied that the game was still too hard to play, and some of the students responded that the period of playing was too short to fully understand binary conversion.

89% of students in EG2 indicated that they learned more about binary conversion by playing and competing with their peers. When they encountered difficulties, the competitor would share the information through the game even if they were competing. This was impressive and learners were more engaged by playing the game.

3. Do you think this method of using an application to learn binary conversion could make learning computer science more interesting? Why?

76% of students in EG1 indicated that it was more interesting to learn by using the application. Some of the students wanted to gain more knowledge about computer science because of understanding the operation of a computer. Few students thought that the course was beyond their capability of comprehension, so they were not interested in learning it.

79% of students in EG2 said that learning computer science by playing a digital game and competing with others was very interesting and exciting. Through their sense of accomplishment, they were more willing to learn computer science.

4. Do you think this method of using an application to learn binary conversion could make you more confident in learning computer science? Why?

75% of students in EG1 expressed that through this course, they had more confidence in learning computer science. Some students gained confidence due to understanding binary conversion through the digital game, some students changed their thoughts about the difficulty of computer science. A few students thought that even though they understood the concept of binary conversion, there was more unknown information in computer science that they did not have the confidence to realize.

81% of students in EG2 indicated they were more confident after learning binary conversion by playing the digital game and competing with their peers. Some of them gained confidence by winning the game, and some of them had more confidence because they fully understood the concept of binary conversion. Few students thought they were not good at computer science and became afraid of not learning as well as the other members of the group.

## Conclusion and Discussion

### The Research Model

#### Factors Under SCT

Studies have shown that students with positive emotions tend to have more support from others (Lent et al., 2017). On the other hand, students with negative emotions do not have as much support as those with positive emotions (Franco et al., 2019). However, some studies have shown that a negative emotional state might not affect students’ social support (Sheu et al., 2017; Chervonsky and Hunt, 2018). The result of this study is the same as that of Sheu et al. (2017) and Chervonsky and Hunt (2018). The reason for this result might be because courses on computer science are fewer than other main subjects, and the emotions students felt during the course might not affect the support that they receive from their friends in their daily life. Or maybe students’ social support was not affected by negative emotions, which is a positive outcome for students.

Oranye et al. (2017) show that social support significantly affects students’ learning performance. However, the result of the present study is different. In the overall result or the result in all three different groups, social support does not significantly affect learning performance in this study. This might be because students have friends they can count on, but this might not influence the knowledge of binary conversion that students have learned or their capability of using this knowledge in the real world.

Although the emotional state does not affect learning performance through social support, and social support does not directly influence learning performance, both emotional state and social support affect learning performance through self-efficacy and learning motivation. This indicates the importance of self-efficacy and learning motivation.

This research agrees with that of previous work investigating the behavior of entrepreneurs in a competition, which implied that entrepreneurs prefer to be more aggressive in competitions (Hmieleski and Lerner, 2016; Levine and Rubinstein, 2017), with a high demand for accomplishment motivation (Rauch and Frese, 2007; Urbig et al., 2020), and that they are more likely to facilitate competitive over non-competitive environments (Bönte and Piegeler, 2013; Urbig et al., 2020).

#### Self-Efficacy and the Factors Under SCT

When students feel stressed, nervous, or depressed, they tend to have lower confidence in learning. On the contrary, when students do not have so many negative emotions, they are likely to have higher self-efficacy (Hong et al., 2019b; García-Pérez et al., 2020). This result is the same as those of previous studies. It demonstrates that when students are not depressed or nervous about learning computer science, they have faith in their capability of binary conversion.

Sheu et al. (2017) have shown that when students have higher social support, they have more confidence in themselves. This result agrees with that of Sheu et al. (2017), indicating that when students have peers or friends from whom they can seek help, they have more confidence in learning binary conversion.

Studies have shown that when students have higher self-efficacy in the environment of online learning, they tend to have higher learning performance (Zhao and Huang, 2020). Hayat et al. (2020) found that students’ self-efficacy and learning performance were positively correlated, and students had an optimistic attitude to learning and teaching materials. Shen et al. (2021) also presented findings that indicated that entrepreneurial self-efficacy completely mediates the relationship between entrepreneurial learning and firm performance. This is the same result as Hayat et al. (2020), Zhao and Huang (2020), and Shen et al. (2021) in this study. If students have more faith in learning binary conversion, they have better learning performance.

#### Emotional State and Learning Motivation

When students are nervous or depressed in learning, they have lower motivation (Kunanitthaworn et al., 2018). Wei et al. (2020) claim that entrepreneurial self-efficacy affects the attitude and behavior of entrepreneurs. This research indicated that entrepreneurs with higher self-efficacy experienced higher job satisfaction, showing a better mental state, with less pressure and emotional exhaustion, which promotes innovation behavior. The results of this study are similar, which means students have higher learning motivation and are more willing to learn computer science when they are not stressed.

#### Self-Efficacy and Learning Motivation

Dissanayake et al. (2019) showed that when students have higher self-efficacy, they have more learning interests and motivation no matter whether they are in a competitive environment or not. The result in this study shows the same. When students have higher self-efficacy in learning binary conversion, they show more interest and have higher learning motivation.

#### Learning Motivation and Learning Performance

When students feel interested or motivated, they have higher learning performance whether in the environment of online learning or using game systems. Learning motivation positively significantly affects learning performance (Zhao and Huang, 2020). The same result has been found in this study, which means students could have better performance when undertaking binary conversion if they feel motivated in learning computer science.

### Learning Achievement

The purpose of this study was to investigate the effect of different teaching methods and environments on students’ learning achievements. The results show that students using digital game-based learning in a competitive environment (EG2) had higher learning achievement than the other two groups (EG1, CG). Two groups using digital game-based learning (EG1, EG2) had higher learning achievements than the students who only learned by conventional approaches (CG; Lin et al., 2018), supporting the following findings:

1. Through digital game-based learning, students have better learning achievement compared to conventional learning approaches.
2. Through the integration of digital game-based learning and a competitive environment, students have higher learning achievements compared to students who learned with only digital game-based learning.

### Interview

We undertook a semi-structured interview after the experiment to gain an understanding of the students’ perception of the content of the course and teaching materials. Through the interview feedback, we aim to improve the content of the course. The results are listed below:

1. Compared with a conventional learning approach, students prefer learning with digital games.
2. Most of the students thought using digital game-based learning could improve their interest and confidence in learning.
3. A significant number of students felt that using digital game-based learning could make their learning more efficient.
4. Some students suggested that they would enjoy learning with digital games more often.

### Contribution

Although digital game-based learning had been investigated frequently, few studies have combined research with a theoretical model. At the same time, this study used SCT to investigate the influences of personal, environmental factors, and behavior. After the verification and analysis, we contribute the following:

1. Research into the combination of SCT and digital game-based learning.
2. The research focuses on courses in computer science.
3. It discovered the importance of self-efficacy and learning motivation.
4. The study advocates for the wider utilization of digital game-based learning.
5. The study offers teachers and researchers references for designing the course and teaching materials.

We expanded research in digital game-based learning by combining SCT and computer science in the situation of competition. Teachers could design the course by implementing digital game-based learning and competition, which might help students improve their learning performance.

### Limitations and Future Works

We have strived to be complete and rigorous in the research framework and experimental design, but there are still many situations and conditions that have not been fully considered in this research. Countries are having issues of low birth rate, and so is Taiwan. The design of this study expected to have three groups of students, which would require more participants than the number of students studying in a single school. We had to collect students from different schools to have adequate numbers for the experiment. Students in different schools might be slightly different, which might affect the results.

Every student in EG1 was equipped with a table or a cellphone, and every two students in EG2 shared one table or one cellphone, which means the requirement for devices was numerous. Therefore, researchers should pay more attention to the amount of equipment while designing future experiments.

The content of this study was the concept of binary conversion. There are more concepts or units in computer science, and it is suggested that future experiments could be extended to different units. It is also suggested that the experiment be implemented on students of different ages since the present study only focused on junior high school students. Furthermore, with the combination of SCT, there are more factors such as cognitive, meta-cognitive, family support, or learning engagement to discuss. In addition, this study mainly investigates the effect of digital game-based learning with a competitive environment. Due to the small amount of research on computer science, other teaching methods such as a flipped classroom, problem-based learning, or augmented reality are suggested for future research. We hope more research on computer science will be further developed in the future.

## Data Availability Statement

The raw data supporting the conclusions of this article will be made available by the authors, without undue reservation.

## Author Contributions

C-CC: research topic and the methodology, research model, statistical analysis, research findings. H-YT: statistical analysis, research writing. All authors contributed to the article and approved the submitted version.

## Funding

The authors wish to thank the Ministry of Science and Technology of the Republic of China for financially supporting this research under Contract Grants No. MOST 108-2511-H-005-001-MY3.

## Conflict of Interest

The authors declare that the research was conducted in the absence of any commercial or financial relationships that could be construed as a potential conflict of interest.

## Publisher’s Note

All claims expressed in this article are solely those of the authors and do not necessarily represent those of their affiliated organizations, or those of the publisher, the editors and the reviewers. Any product that may be evaluated in this article, or claim that may be made by its manufacturer, is not guaranteed or endorsed by the publisher.

## Appendix 1

**Table 1:**

Items label	Initial items
Section 1	
ES1	Just being in computer science class makes me feel stressed and nervous.
ES2	Doing computer practical activity takes all of my energy.
ES3	I start to feel stressed-out as soon as I begin my computer practical activity.
ES4	I get depressed when I think about learning computer science.
SE1	I'm certain I can understand the most difficult material presented in the readings for this course.
SE2	I'm confident I can understand the basic concepts taught in this course.
SE3	I'm confident I can understand the most complex material presented by the instructor in this course.
SE4	I expect to do well in this class.
SE5	Considering the difficulty of this course, I think I will do well in this class.
SS1	There are several people that I trust to help solve my problems.
SS2	There is no one that I feel comfortable to talking about intimate personal problems.
SS3	When I need suggestions on how to deal with a personal problem, I know someone I can turn to.
SS4	There is at least one person I know whose advice I really trust.
LM1	Learning computer science is interesting.
LM2	I am curious about discoveries in computer science.
LM3	The computer science I learn is relevant to my life.
LM4	Learning computer science makes my life more meaningful.
LM5	I enjoy learning computer science.
LP1	After the course, I have increased knowledge of binary conversion.
LP2	After the course, I could apply knowledge to practical situation.
LP3	After the course, I have more confidence in dealing with binary conversion problems.
LP4	After the course, I could gain experience and competence of problem-solving.
Section 2					
1. Class:	
2. Gender: M/F	

## References

[ref1] Almås I. Cappelen A. W. Salvanes K. G. Sorensen E. O. Tungodden B. (2016). What explains the gender gap in college track dropout? Experimental and administrative evidence. Am. Econ. Rev. 106, 296–302. doi: 10.1257/aer.p20161075

[ref2] Arguedas M. Daradoumis A. Xhafa Xhafa F. (2016). Analyzing how emotion awareness influences students’ motivation, engagement, self-regulation and learning outcome. Educ. Technol. Soc. 19, 87–103.

[ref3] Bagozzi R. P. Yi Y. (1988). On the evaluation of structural equation models. J. Acad. Market Sci. 16, 74–94. doi: 10.1007/BF02723327

[ref4] Bandura A. (1977). Self-efficacy: toward a unifying theory of behavioral change. Psychol. Rev. 84, 191–215. doi: 10.1037/0033-295X.84.2.191, PMID: 847061

[ref6] Bandura A. (1989). “Social cognitive theory,” in Annals of Child Development. Vol. 6. Six Theories of Child Development. ed. Vasta R. (Greenwich: JAI Press), 1–60.

[ref7] Baum W. M. (2017). Understanding Behaviorism: Behavior, Culture, and Evolution. John Wiley and Sons.

[ref8] Bönte W. Piegeler M. (2013). Gender gap in latent and nascent entrepreneurship: driven by competitiveness. Small Bus. Econ. 41, 961–987. doi: 10.1007/s11187-012-9459-3

[ref9] Burnett A. J. Smith T. M. E. Wessel M. T. (2016). Use of the social cognitive theory to frame university students’ perceptions of cheating. J. Acad. Ethics 14, 49–69. doi: 10.1007/s10805-015-9252-4

[ref10] Buser T. Peter N. Wolter S. C. (2017). Gender, competitiveness, and study choices in high school: evidence from Switzerland. Am. Econ. Rev. 107, 125–130. doi: 10.1257/aer.p20171017

[ref11] Cagiltay N. E. Ozcelik E. Ozcelik N. S. (2015). The effect of competition on learning in games. Comput. Educ. 87, 35–41. doi: 10.1016/j.compedu.2015.04.001

[ref12] Cardon M. S. Kirk C. P. (2015). Entrepreneurial passion as mediator of the self-efficacy to persistence relationship. Entrep. Theory Pract. 39, 1027–1050. doi: 10.1111/etap.12089

[ref13] Chen C. H. (2019). The impacts of peer competition-based science gameplay on conceptual knowledge, intrinsic motivation, and learning behavioral patterns. Educ. Technol. Res. Dev. 67, 179–198. doi: 10.1007/s11423-018-9635-5

[ref14] Chen C. C. Huang P. H. (2020). The effects of STEAM-based mobile learning on learning achievement and cognitive load. Interact. Learn. Environ., 1–17. doi: 10.1080/10494820.2020.1761838

[ref15] Chen C. H. Law V. Huang K. (2019). The roles of engagement and competition on learner’s performance and motivation in game-based science learning. Educ. Technol. Res. Dev. 67, 1003–1024. doi: 10.1007/s11423-019-09670-7

[ref16] Chervonsky E. Hunt C. (2018). Emotion suppression and reappraisal associated with bullying involvement and other social outcomes in young adults. Soc. Psychol. Educ. 21, 849–873. doi: 10.1007/s11218-018-9440-3

[ref17] Cohen S. Mermelstein R. Kamarck T. Hoberman H. M. (1985). “Measuring the functional components of social support,” in Social Support: Theory, Research and Applications (Dordrecht: Springer), 73–94.

[ref18] Deng L. Wu S. Chen Y. Peng Z. (2020). Digital game-based learning in a Shanghai primary-school mathematics class: a case study. J. Comput. Assist. Learn. 36, 709–717. doi: 10.1111/jcal.12438

[ref19] Dissanayake I. Mehta N. Palvia P. Taras V. Amoako-Gyampah K. (2019). Competition matters! Self-efficacy, effort, and performance in crowdsourcing teams. Inf. Manage. 56:103158. doi: 10.1016/j.im.2019.04.001

[ref20] Eisenberg J. Thompson W. F. (2011). The effects of competition on improvisers’ motivation, stress, and creative performance. Creat. Res. J. 23, 129–136. doi: 10.1080/10400419.2011.571185

[ref900] Fidan M. Tuncel M. (2019). Integrating augmented reality into problem based learning: The effects on learning achievement and attitude in physics education. Comp. Educ. 142: 103635. doi: 10.1016/j.compedu.2019.103635

[ref21] Ford M. E. (1992). Human Motivation: Goals, Emotions, and Personal Agency Beliefs. Newbury Park, CA: Sage.

[ref22] Fornell C. Larcker D. F. (1981). Sturctural equation models with unobservable variables and measurement error: algebra and statistics. J. Mark. Res. 18, 382–388. doi: 10.1177/002224378101800313

[ref23] Franco M. Hsiao Y. S. Gnilka P. B. Ashby J. S. (2019). Acculturative stress, social support, and career outcome expectations among international students. Int. J. Educ. Vocat. Guid. 19, 275–291. doi: 10.1007/s10775-018-9380-7

[ref24] García-Pérez O. Inda-Caro M. Fernández-García C. M. Torío-López S. (2020). The influence of perceived family supports and barriers on personal variables in a Spanish sample of secondary school science-technology students. Int. J. Sci. Educ. 42, 70–88. doi: 10.1080/09500693.2019.1701216

[ref25] Glynn S. M. Brickman P. Armstrong N. Taasoobshirazi G. (2011). Science motivation questionnaire II: validation with science majors and nonscience majors. J. Res. Sci. Teach. 48, 1159–1176. doi: 10.1002/tea.20442

[ref26] Hair J. F. Black W. C. Babin B. J. Anderson R. E. Tatham R. L. (1998). Multivariate Data Analysis. 5th *edn*. Upper Saddle River, NJ: Prentice Hall.

[ref27] Hayat A. A. Shateri K. Amini M. Shokrpour N. (2020). Relationships between academic self-efficacy, learning-related emotions, and metacognitive learning strategies with academic performance in medical students: a structural equation model. BMC Med. Educ. 20:76. doi: 10.1186/s12909-020-01995-9, PMID: 32183804 PMC7079530

[ref28] Hmieleski K. M. Lerner D. A. (2016). The dark triad and nascent entrepreneurship: an examination of unproductive versus productive entrepreneurial motives. J. Small Bus. Manage. 54, 7–32. doi: 10.1111/jsbm.12296

[ref29] Hong J. C. Hwang M. Y. Tai K. H. Lin P. H. (2019a). Improving cognitive certitude with calibration mediated by cognitive anxiety, online learning self-efficacy and interest in learning Chinese pronunciation. Educ. Technol. Res. Dev. 67, 597–615. doi: 10.1007/s11423-018-9628-4

[ref30] Hong J. C. Ye J. H. Shih Y. Y. (2019b). Positive affect creative self-efficacy on the ability and confidence to predict problem solving avoidance motivation in a digital advertisement design course. Bull. Educ. Psychol. 51, 321–339. doi: 10.6251/BEP.201912_51(2).0007

[ref31] Hung C. M. Huang I. Hwang G. J. (2014). Effects of digital game-based learning on students’ self-efficacy, motivation, anxiety, and achievements in learning mathematics. J. Comput. Educ. 1, 151–166. doi: 10.1007/s40692-014-0008-8

[ref32] Johnsen B. H. Espevik R. Saus E. R. Sanden S. Olsen O. K. Hystad S. W. (2017). Hardiness as a moderator and motivation for operational duties as mediator: the relation between operational self-efficacy, performance satisfaction, and perceived strain in a simulated police training scenario. J. Police Crim. Psychol. 32, 331–339. doi: 10.1007/s11896-017-9225-1

[ref33] Johnson D. W. Johnson R. T. (2009). An educational psychology success story: social interdependence theory and cooperative learning. Educ. Res. 38, 365–379. doi: 10.3102/0013189X09339057

[ref34] Joo Y. J. Oh E. Kim S. M. (2015). Motivation, instructional design, flow, and academic achievement at a Korean online university: a structural equation modeling study. J. Comput. High. Educ. 27, 28–46. doi: 10.1007/s12528-015-9090-9

[ref35] King R. B. Ganotice F. A. (2014). The social underpinnings of motivation and achievement: investigating the role of parents, teachers, and peers on academic outcomes. Asia-Pac. Educ. Res. 23, 745–756. doi: 10.1007/s40299-013-0148-z

[ref36] Kirzner I. M. (1973). Competition and Entrepreneurship. Chicago, London: The University of Chicago Press.

[ref37] Kunanitthaworn N. Wongpakaran T. Wongpakaran N. Paiboonsithiwong S. Songtrijuck N. Kuntawong P.. (2018). Factors associated with motivation in medical education: a path analysis. BMC Med. Educ. 18:140. doi: 10.1186/s12909-018-1256-5, PMID: 29914462 PMC6006981

[ref38] Lent R. W. Ezeofor I. Morrison M. A. Penn L. T. Ireland G. W. (2016). Applying the social cognitive model of career self-management to career exploration and decision-making. J. Vocat. Behav. 93, 47–57. doi: 10.1016/j.jvb.2015.12.007

[ref39] Lent R. W. Taveira M. D. C. Figuera P. Dorio I. Faria S. Gonçalves A. M. (2017). Test of the social cognitive model of well-being in Spanish college students. J. Career Assess. 25, 135–143. doi: 10.1177/1069072716657821

[ref40] Levine R. Rubinstein Y. (2017). Smart and illicit: who becomes an entrepreneur and do they earn more? Q. J. Econ. 132, 963–1018. doi: 10.1093/qje/qjw044

[ref41] Li L. K. (2012). A study of the attitude, self-efficacy, effort and academic achievement of CityU students towards research methods and statistics. Discov. SS Stud. E J. 1, 154–183.

[ref42] Lim S. Oliver M. (2015). A Guide to the International Biology Olympiad. IBO Coordinating Center, Prague.

[ref43] Lin H. C. Chang C. M. (2018). What motivates health information exchange in social media? The roles of the social cognitive theory and perceived interactivity. Inf. Manage. 55, 771–780. doi: 10.1016/j.im.2018.03.006

[ref44] Lin Y. C. Hsieh Y. H. Hou H. T. Wang S. M. (2019). Exploring students’ learning and gaming performance as well as attention through a drill-based gaming experience for environmental education. J. Comput. Educ. 6, 315–334. doi: 10.1007/s40692-019-00130-y

[ref45] Lin C. J. Hwang G. J. Fu Q. K. Chen J. F. (2018). A flipped contextual game-based learning approach to enhancing EFL students’ English business writing performance and reflective behaviors. Educ. Technol. Soc. 21, 117–131.

[ref46] Lin T. J. Tsai C. C. (2018). Differentiating the sources of Taiwanese high school students’ multidimensional science learning self-efficacy: an examination of gender differences. Res. Sci. Educ. 48, 575–596. doi: 10.1007/s11165-016-9579-x

[ref47] Liu Y. C. Hung Y. Y. (2016). Self-efficacy as the moderator: exploring driving factors of perceived social support for mainland Chinese students in Taiwan. Comput. Hum. Behav. 64, 455–462. doi: 10.1016/j.chb.2016.07.018

[ref48] Liu K. P. Tai S. J. D. Liu C. C. (2018). Enhancing language learning through creation: the effect of digital storytelling on student learning motivation and performance in a school English course. Educ. Technol. Res. Dev. 66, 913–935. doi: 10.1007/s11423-018-9592-z

[ref49] Niederle M. (2017). A gender agenda: a progress report on competitiveness. Am. Econ. Rev. 107, 115–119. doi: 10.1257/aer.p20171066

[ref50] Obschonka M. Moeller J. Goethner M. (2019). Entrepreneurial passion and personality: the case of academic entrepreneurship. Front. Psychol. 9:2697. doi: 10.3389/fpsyg.2018.02697, PMID: 30687165 PMC6335975

[ref51] Oranye N. O. Ezeah P. Ahmad N. (2017). Elements of social capital and academic performance of undergraduate students. Soc. Indic. Res. 131, 305–319. doi: 10.1007/s11205-016-1249-x

[ref52] Özhan Ş. Ç. Kocadere S. A. (2020). The effects of flow, emotional engagement, and motivation on success in a gamified online learning environment. J. Educ. Comput. Res. 57, 2006–2031. doi: 10.1177/0735633118823159

[ref53] Phan H. P. (2016). Longitudinal examination of optimism, personal self-efficacy and student well-being: a path analysis. Soc. Psychol. Educ. 19, 403–426. doi: 10.1007/s11218-015-9328-4

[ref54] Pintrich P. R. Smith D. A. F. Garcia T. McKeachie W. J. (1991). A Manual for the Use of the Motivated Strategies for Learning Questionnaire (MSLQ) (Ann Arbor, MI: National Center for Research to Improve Postsecondary Teaching and Learning), 1–76.

[ref55] Pumptow M. Brahm T. (2021). Students’ digital media self-efficacy and its importance for higher education institutions: development and validation of a survey instrument. Technol. Knowl. Learn. 26, 555–575. doi: 10.1007/s10758-020-09463-5

[ref56] Rauch A. Frese M. (2007). Let’s put the person back into entrepreneurship research: a meta-analysis on the relationship between business owners’ personality traits, business creation, and success. Eur. J. Work Organ. Psy. 16, 353–385. doi: 10.1080/13594320701595438

[ref57] Reuben E. Wiswall M. Zafar B. (2017). Preferences and biases in educational choices and labour market expectations: shrinking the black box of gender. Econ. J. 127, 2153–2186. doi: 10.1111/ecoj.12350

[ref58] Ryan R. M. Deci E. L. (2000). Intrinsic and extrinsic motivations: classic definitions and new directions. Contemp. Educ. Psychol. 25, 54–67. doi: 10.1006/ceps.1999.1020, PMID: 10620381

[ref59] Sandberg J. Maris M. Hoogendoorn P. (2014). The added value of a gaming context and intelligent adaptation for a mobile learning application for vocabulary learning. Comput. Educ. 76, 119–130. doi: 10.1016/j.compedu.2014.03.006

[ref60] Schumpeter J. A. (1934). The Theory of Economic Development. Cambridge, MA: Harvard University Press.

[ref61] Shen Y. Wang Q. Hua D. Zhang Z. (2021). Entrepreneurial learning, self-efficacy, and firm performance: exploring moderating effect of entrepreneurial orientation. Front. Psychol. 12:731628. doi: 10.3389/fpsyg.2021.731628, PMID: 34512486 PMC8426342

[ref62] Sheu H. B. Liu Y. Li Y. (2017). Well-being of college students in China: testing a modified social cognitive model. J. Career Assess. 25, 144–158. doi: 10.1177/1069072716658240

[ref63] Su C. H. (2016). The effects of students’ motivation, cognitive load and learning anxiety in gamification software engineering education: a structural equation modeling study. Multimed. Tools Appl. 75, 10013–10036. doi: 10.1007/s11042-015-2799-7

[ref64] Sun J. C. Y. Chang K. Y. Chen Y. H. (2015). GPS sensor-based mobile learning for English: an exploratory study on self-efficacy, self-regulation and student achievement. Res. Pract. Technol. Learn. 10:23. doi: 10.1186/s41039-015-0024-y, PMID: 30613232 PMC6302839

[ref65] ter Vrugte J. de Jong T. Vandercruysse S. Wouters P. van Oostendorp H. Elen J. (2015). How competition and heterogeneous collaboration interact in prevocational game-based mathematics education. Comp. Educ. 89, 42–52. doi: 10.1016/j.compedu.2015.08.010

[ref66] Tian Y. Fang Y. Li J. (2018). The effect of metacognitive knowledge on mathematics performance in self-regulated learning framework-multiple mediation of self-efficacy and motivation. Front. Psychol. 9:2518. doi: 10.3389/fpsyg.2018.02518, PMID: 30631293 PMC6315178

[ref67] Tokan M. K. Imakulata M. M. (2019). The effect of motivation and learning behaviour on student achievement. S. Afr. J. Educ. 39:1510. doi: 10.15700/saje.v39n1a1510

[ref68] Uçar F. M. Sungur S. (2017). The role of perceived classroom goal structures, self-efficacy, and engagement in student science achievement. Res. Sci. Technol. Educ. 35, 149–168. doi: 10.1080/02635143.2017.1278684

[ref69] Urbig D. Bönte W. Procher V. D. Lombardo S. (2020). Entrepreneurs embrace competition: evidence from a lab-in-the-field study. Small Bus. Econ. 55, 193–214. doi: 10.1007/s11187-019-00141-0

[ref70] Usher E. L. Pajares F. (2009). Sources of self-efficacy in mathematics: a validation study. Contemp. Educ. Psychol. 34, 89–101. doi: 10.1016/j.cedpsych.2008.09.002

[ref71] Vogl K. Preckel F. (2014). Full-time ability grouping of gifted students impacts on social self-concept and school-related attitudes. Gifted Child Q. 58, 51–68. doi: 10.1177/0016986213513795

[ref72] Wang J. H. Gamble J. H. Yang C. (2020). Mobile sensor-based community gaming for improving vocational students’ sleep and academic outcomes. Comp. Educ. 151:103812. doi: 10.1016/j.compedu.2020.103812

[ref73] Wang X. Wallace M. P. Wang Q. (2017). Rewarded and unrewarded competition in a CSCL environment: a coopetition design with a social cognitive perspective using PLS-SEM analyses. Comput. Hum. Behav. 72, 140–151. doi: 10.1016/j.chb.2017.02.045

[ref74] Wei J. Chen Y. Zhang Y. Zhang J. (2020). How does entrepreneurial self-efficacy influence innovation behavior? Exploring the mechanism of job satisfaction and zhongyong thinking. Front. Psychol. 11:708. doi: 10.3389/fpsyg.2020.00708, PMID: 32457676 PMC7227373

[ref75] Woodworth R. S. (1918). Columbia University Lectures: Dynamic Psychology. New York: Columbia University Press.

[ref76] Wu H.-T. Chen M.-Y. (2019). Course design for college entrepreneurship education – from personal trait analysis to operation in practice. Front. Psychol. 10:1016. doi: 10.3389/fpsyg.2019.01016, PMID: 31231261 PMC6558313

[ref77] Wu W.-H. Kao H.-Y. Wu S.-H. Wei C.-W. (2019). Development and evaluation of affective domain using student’s feedback in entrepreneurial massive open online courses. Front. Psychol. 10:1109. doi: 10.3389/fpsyg.2019.01109, PMID: 31178782 PMC6542944

[ref78] Yang J. C. Chen S. Y. (2020). An investigation of game behavior in the context of digital game-based learning: an individual difference perspective. Comput. Hum. Behav. 112:106432. doi: 10.1016/j.chb.2020.106432

[ref79] Yueh H.-P. Wu Y. J. Chen W.-F. (2020). Editorial: the psychology and education of entrepreneurial development. Front. Psychol. 11:27. doi: 10.3389/fpsyg.2020.00027, PMID: 32038440 PMC6992604

[ref80] Zhan Y. Jiang Y. Wan Z. H. Guo J. J. (2021). Is there an “expectancy× value” effect? Investigating the impact of self-efficacy and learning motives on Chinese undergraduates’ use of deep language learning strategies. Asia-Pac. Educ. Res. 30, 83–94. doi: 10.1007/s40299-020-00516-y

[ref81] Zhao Q. Huang X. (2020). Individual differences in response to attributional praise in an online learning environment. Educ. Technol. Res. Dev. 68, 1069–1087. doi: 10.1007/s11423-019-09720-0

