# Supplementary material for: The Effect of Digital Game-Based Learning on Learning Motivation and Performance Under Social Cognitive Theory and Entrepreneurial Thinking
Source: Front Psychol. 2021 Dec 16;12:750711. doi: 10.3389/fpsyg.2021.750711 (PMC8716945; doi:10.3389/fpsyg.2021.750711)
Supplement: Supplementary file 1 [file Table_1.DOCX]

**Table 1**

Demographic details of respondents.

| Group | Control group | Experimental group 1 | Experimental group 2 | Total |
| --- | --- | --- | --- | --- |
| Sample | (CG) | (EG1) | (EG2) |  |
| Valid samples | 173 | 143 | 194 | 510 |
| Invalid samples | 5 | 48 | 19 | 72 |
| Total | 178 | 191 | 213 | 582 |

**Table 2**

Reliability index.

| Latent variable | Manifest variables (MVs) | Cronbach’s alpha |
| --- | --- | --- |
| Emotional state (ES) | 4 | 0.890 |
| Social support (SS) | 3 | 0.770 |
| Self-efficacy (SE) | 4 | 0.874 |
| Learning motivation (LM) | 5 | 0.925 |
| Learning performance (LP) | 4 | 0.899 |

**Table 3**

The results of confirmatory factor Analysis: Factor loadings, CR, and AVE.

| Latent variable | MVs | Factor loadings | CR | AVE |
| --- | --- | --- | --- | --- |
| Emotional state (ES) | ES1 | 0.828 | 0.9096 | 0.7157 |
|  | ES2 | 0.838 |  |  |
|  | ES3 | 0.835 |  |  |
|  | ES4 | 0.882 |  |  |
| Social support (SS) | SS1 | 0.737 | 0.8632 | 0.6789 |
|  | SS3 | 0.880 |  |  |
|  | SS4 | 0.848 |  |  |
| Self-efficacy (SE) | SE1 | 0.856 | 0.8413 | 0.5735 |
|  | SE2 | 0.769 |  |  |
|  | SE3 | 0.777 |  |  |
|  | SE5 | 0.605 |  |  |
| Learning motivation (LM) | LM1 | 0.784 | 0.9065 | 0.6602 |
|  | LM2 | 0.854 |  |  |
|  | LM3 | 0.765 |  |  |
|  | LM4 | 0.812 |  |  |
|  | LM5 | 0.844 |  |  |
| Learning performance (LP) | LP1 | 0.790 | 0.8314 | 0.5527 |
|  | LP2 | 0.684 |  |  |
|  | LP3 | 0.748 |  |  |
|  | LP4 | 0.748 |  |  |

**Table 4**

Correlation among construct scores

|  | Mean | SD | ES | SS | SE | LM | LP |
| --- | --- | --- | --- | --- | --- | --- | --- |
| ES | 2.00 | 0.95 | **0.846** |  |  |  |  |
| SS | 3.85 | 0.94 | -0.070 | **0.824** |  |  |  |
| SE | 3.04 | 0.99 | -0.223 | 0.164 | **0.757** |  |  |
| LM | 3.42 | 1.02 | -0.413 | 0.198 | 0.458 | **0.813** |  |
| LP | 3.17 | 0.99 | -0.242 | 0.185 | 0.736 | 0.606 | **0.743** |

Note: The boldface figures in the diagonal represent the square root of AVE figures.

**Table 5**

Model fit.

| Fit indices |  | Recommended value | SEM results | Reference |
| --- | --- | --- | --- | --- |
| Absolute fit indices | $\chi^{2}$/df | <3 | 2.772 | Hair et al. (1998) |
|  | GFI | >0.8 | 0.916 | Bagozzi & Yi (1988)  Browne & Cudec (1992) |
|  | AGFI | >0.8 | 0.891 | Bagozzi & Yi (1988)  Segars & Grover (1993) |
|  | RMSEA | <0.08 | 0.059 | Hair et al. (1998) |
| Incremental fit indices | NFI | >0.9 | 0.936 | Hair et al. (1998) |
|  | RFI | >0.9 | 0.925 | Hair et al. (1998) |
|  | IFI | >0.9 | 0.958 | Hair et al. (1998) |
|  | NNFI | >0.9 | 0.951 | Hair et al. (1998) |
|  | CFI | >0.9 | 0.958 | Hair et al. (1998) |
| Parsimonious fit indices | PNFI | >0.5 | 0.798 | Bagozzi & Yi (1988) |
|  | PGFI | >0.5 | 0.707 | Bagozzi & Yi (1988) |
|  | PCFI | >0.5 | 0.817 | Bagozzi & Yi (1988) |

**Table 6**

Structural path coefficients.

|  | SE |  | LM |  | LP |  |
| --- | --- | --- | --- | --- | --- | --- |
|  | Direct | Indirect | Direct | Indirect | Direct | Indirect |
| ES | -0.218*** |  | -0.370*** | -0.090 |  | -0.292 |
| SS | 0.154** |  |  | 0.063 |  | 0.122 |
| SE |  |  | 0.412*** |  | 0.661*** | 0.132 |
| LM |  |  |  |  | 0.321*** |  |

**Table 7**

Hypotheses justification.

| H | Path | *β* | C.R. | *p* | Decision |
| --- | --- | --- | --- | --- | --- |
| H1(-) | Emotional state → Learning motivation | -0.370 | -8.692 | <0.001 | Supported |
| H2(-) | Emotional state → Self-efficacy | -0.218 | -4.455 | <0.001 | Supported |
| H3(-) | Emotional state → Social support | -0.077 | -1.501 | 0.133 | Not supported |
| H4 | Self-efficacy → Learning motivation | 0.412 | 9.088 | <0.001 | Supported |
| H5 | Social support → Self-efficacy | 0.154 | 3.009 | 0.003 | Supported |
| H6 | Learning motivation → Learning performance | 0.321 | 8.662 | <0.001 | Supported |
| H7 | Self-efficacy → Learning performance | 0.661 | 13.347 | <0.001 | Supported |
| H8 | Social support → Learning performance | 0.021 | 0.666 | 0.506 | Not supported |

**Table 8**

Pretest ANOVA analysis.

| Group | Mean | SD | F | *p* |
| --- | --- | --- | --- | --- |
| CG | 44.49 | 13.572 | 1.768 | .172 |
| EG1 | 44.76 | 13.834 |  |  |
| EG2 | 42.30 | 15.748 |  |  |
| **p*<.05, ***p*<.01, ****p*<.001 | | | | |

**Table 9**

Pretest pairwise comparison.

| Group (I) | Group (J) | Mean difference (I-J) | SD Error | *p* |
| --- | --- | --- | --- | --- |
| CG | EG1 | -0.27 | 1.428 | .850 |
|  | EG2 | 2.194 | 1.483 | .140 |
| EG1 | CG | 0.27 | 1.428 | .850 |
|  | EG2 | 2.464 | 1.472 | .095 |
| EG2 | CG | -2.194 | 1.483 | .140 |
|  | EG1 | -2.464 | 1.472 | .095 |
| **p*<.05, ***p*<.01, ****p*<.001 | | | | |

**Table 10**

Posttest ANOVA analysis.

| Group | Mean | SD | F | *p* |
| --- | --- | --- | --- | --- |
| CG | 49.83 | 13.297 | 13.816 | .000*** |
| EG1 | 54.19 | 17.298 |  |  |
| EG2 | 57.79 | 13.849 |  |  |
| **p*<.05, ***p*<.01, ****p*<.001 | | | | |

**Table 11**

Posttest pairwise comparison.

| Group (I) | Group (J) | Mean difference (I-J) | SD Error | *p* |
| --- | --- | --- | --- | --- |
| CG | EG1 | -4.357 | 1.600 | .007** |
|  | EG2 | -7.962 | 1.381 | .000*** |
| EG1 | CG | 4.357 | 1.600 | .007** |
|  | EG2 | -3.605 | 1.571 | .022* |
| EG2 | CG | 7.962 | 1.381 | .000*** |
|  | EG1 | 3.605 | 1.571 | .022* |
| **p*<.05, ***p*<.01, ****p*<.001 | | | | |
